# Risk factors for pneumonitis in patients with non‐small cell lung cancer treated with immune checkpoint inhibitors plus chemotherapy: A retrospective analysis

**DOI:** 10.1111/1759-7714.14308

**Published:** 2022-01-19

**Authors:** Teppei Yamaguchi, Junichi Shimizu, Yuko Oya, Naohiro Watanabe, Takaaki Hasegawa, Yoshitsugu Horio, Yoshitaka Inaba, Yutaka Fujiwara

**Affiliations:** ^1^ Department of Thoracic Oncology Aichi Cancer Center Hospital Nagoya Aichi Japan; ^2^ Department of Diagnostic and Interventional Radiology Aichi Cancer Center Hospital Nagoya Aichi Japan

**Keywords:** immune checkpoint inhibitors, interstitial lung disease, non‐small cell lung cancer, pemetrexed, pneumonitis

## Abstract

**Background:**

Immune checkpoint inhibitor (ICI) therapy plus chemotherapy has become a standard of care for patients with advanced non‐small cell lung cancer (NSCLC). Pre‐existing interstitial lung disease (ILD) is a risk factor for drug‐induced pneumonitis caused by chemotherapy or ICI monotherapy. However, clinical data in patients with pre‐existing ILD who received ICI therapy plus chemotherapy are limited. This study aimed to identify the risk factors for drug‐induced pneumonitis in patients with NSCLC treated with ICIs plus chemotherapy.

**Methods:**

We retrospectively reviewed the medical records of 160 consecutive patients who were diagnosed with NSCLC and treated with ICIs plus chemotherapy at Aichi Cancer Center Hospital between December 2018 and November 2020. Patients with a prior history of ICI treatment or thoracic radiotherapy were excluded from the analysis.

**Results:**

Among 125 patients, pre‐existing ILD was observed in 20 patients (16.0%). Drug‐induced pneumonitis developed in 17 patients (13.6%), with a median time to onset of 19.3 weeks (range, 1.6–108.9 weeks). In multivariate logistic analysis, pre‐existing ILD (odds ratio = 19.07, *p* = 0.0001) and PEM exposure (odds ratio = 5.67, *p* = 0.022) were identified as risk factors for the development of drug‐induced pneumonitis.

**Conclusions:**

Pre‐existing ILD and pemetrexed exposure are risk factors for drug‐induced pneumonitis in patients with NSCLC.

## Graphical abstract

Immune checkpoint inhibitors (ICIs) plus chemotherapy have become a standard of care for patients with advanced non‐small cell lung cancer (NSCLC). However, clinical data in patients with pre‐existing ILD are limited. We retrospectively reviewed the medical records of 160 consecutive patients who were diagnosed with NSCLC and treated with ICIs plus chemotherapy between December 2018 and November 2020. A total of 125 patients were finally included in the analysis. Drug‐induced pneumonitis developed in 17 patients (13.6%), with a median time to onset of 19.3 weeks (range, 1.6–108.9 weeks). Univariate and multivariate logistic regression analyses revealed that pre‐existing interstitial lung disease (odds ratio = 19.07, *p* = 0.0001) and pemetrexed exposure (odds ratio = 5.67, *p* = 0.022) significantly increased the risk of drug‐induced pneumonitis.

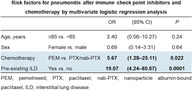

## INTRODUCTION

Immune checkpoint inhibitors (ICIs) including anti‐programmed cell death protein 1 (PD‐1) and anti‐programmed death‐ligand 1 (PD‐L1) antibodies have dramatically improved treatment outcomes in advanced stage non‐small cell lung cancer (NSCLC).^1^, ^2^, ^3^, ^4^, ^5^ ICIs were initially approved for patients who failed first‐line treatment with traditional cytotoxic chemotherapy.^1^, ^2^, ^3^ Subsequent clinical trials demonstrated that the combination of cytotoxic chemotherapy and ICI therapy outperforms platinum‐based chemotherapy in patients with NSCLC in the first‐line setting.^6^, ^7^, ^8^, ^9^, ^10^ However, some patients treated with ICIs develop severe, potentially life‐threatening immune‐related adverse events (irAEs), including pneumonitis.

The incidence of drug‐induced pneumonitis attributable to anti‐PD‐1/PD‐L1 monotherapy has been reported as approximately 5% in previous clinical trials.^1^, ^2^, ^3^, ^4^, ^5^ On the contrary, real‐world reports tended to record higher rates of ICI‐induced pneumonitis of 10%–20%.^11^, ^12^, ^13^, ^14^, ^15^ The increased incidence of ICI‐induced pneumonitis in the real‐world clinical setting is closely related to pre‐existing interstitial lung disease (ILD) or poor performance status, and patients with these features were excluded from clinical trials. In addition, a recent meta‐analysis indicated that ICI‐based regimens were associated with a significantly higher risk of all‐grade and grade ≥ 3 drug‐induced pneumonitis than chemotherapy alone.^16^ However, the association between the onset of drug‐induced pneumonitis attributable to ICI therapy plus chemotherapy and pre‐existing ILD remains unclear. In addition, the risk factors for pneumonitis other than pre‐existing ILD are also unclear.

Hence, this study explored the risk factors for drug‐induced pneumonitis in patients with NSCLC who received ICI therapy plus cytotoxic chemotherapy.

## METHODS

### Patients

Consecutive patients with NSCLC who received ICIs plus cytotoxic chemotherapy at Aichi Cancer Center Hospital between December 2018 and November 2020 were retrospectively analyzed. All patients received one of the following ICI plus chemotherapy regimens: (1) pembrolizumab 200 mg/bodyweight on day 1, cisplatin (CDDP) 60–75 mg/m^2^ or carboplatin (CBDCA) area under the curve (AUC) 4–5 on day 1, and pemetrexed (PEM) 400–500 mg/m^2^ on day 1 for up to four cycles, followed by pembrolizumab plus PEM for maintenance; (2) pembrolizumab 200 mg/bodyweight on day 1, CBDCA AUC 5–6 on day 1, and paclitaxel (PTX) 160–200 mg/m^2^ on day 1 or nanoparticle albumin‐bound paclitaxel (nab‐PTX) 75–100 mg/m^2^ on days 1, 8, and 15 for up to four cycles, followed by pembrolizumab maintenance; (3) atezolizumab 1200 mg/bodyweight on day 1, CBDCA AUC 5–6 on day 1, and nab‐PTX 75–100 mg/m^2^ on days 1, 8, and 15 for up to four cycles, followed by atezolizumab for maintenance; and (4) atezolizumab 1200 mg/bodyweight, bevacizumab (BEV) 15 mg/kg, CBDCA AUC 5–6, and PTX 160–200 mg/m^2^ on day 1 for up to four cycles, followed by atezolizumab plus BEV for maintenance. All treatments were administered intravenously in 3‐week cycles and continued until the appearance of progressive disease or toxicity.

The exclusion criteria were as follows: (1) prior history of thoracic radiotherapy including the lungs, mediastinum, thoracic spine, or ribs; (2) receipt of concurrent or sequential chest radiotherapy; and (3) prior history of ICI therapy. The baseline characteristics of patients including chest computed tomography (CT) findings at the time of treatment initiation were collected from medical records.

### Radiographic analysis

All subjects in our study were examined using a helical CT scanner (1–10 mm slice thickness) immediately before starting ICI plus chemotherapy administration. The radiographic patterns of pre‐existing ILD were assessed by a diagnostic radiologist (TH) and a pulmonologist (JS) in accordance with the Official ATS/ERS/JRS/ALAT Clinical Practice Guideline, and final decisions being reached through consensus.^17^ Pneumonitis was assessed according to the National Cancer Institute Common Toxicity Criteria for Adverse Events, version 5.0.^18^ For patients who developed pneumonitis, date of pneumonitis diagnosis, and maximum grade of pneumonitis were recorded. Pneumonitis was diagnosed by excluding other possible diagnoses and carefully assessing radiographic findings on chest radiographs or CT imaging. Nondrug‐induced pneumonitis, such as that associated with tumor progression, radiation, infection, and pulmonary edema, was excluded by careful investigation.

### Statistical analysis

Progression‐free survival (PFS) was defined as the time from the first day of ICI plus chemotherapy administration to the day of clinical or radiographic disease progression or death. Overall survival (OS) was defined as the time from the first day of treatment to the day of death from any cause. Survival probabilities were estimated using the Kaplan–Meier method and compared using the log‐rank test. The hazard ratio (HR) and 95% confidence interval (CI) were calculated using the univariate Cox proportional hazard model. Univariate and multivariate analyses were conducted using a logistic regression model to evaluate the risk factors of pneumonitis. All variables significant at *p* < 0.2 in the univariate analysis were included in the multivariate analyses. All analyses were performed using JMP pro version 14.30 statistical software (SAS Institute Inc.). The analysis cutoff date was April 30, 2021.

## RESULTS

### Patient characteristics

**FIGURE 1:**
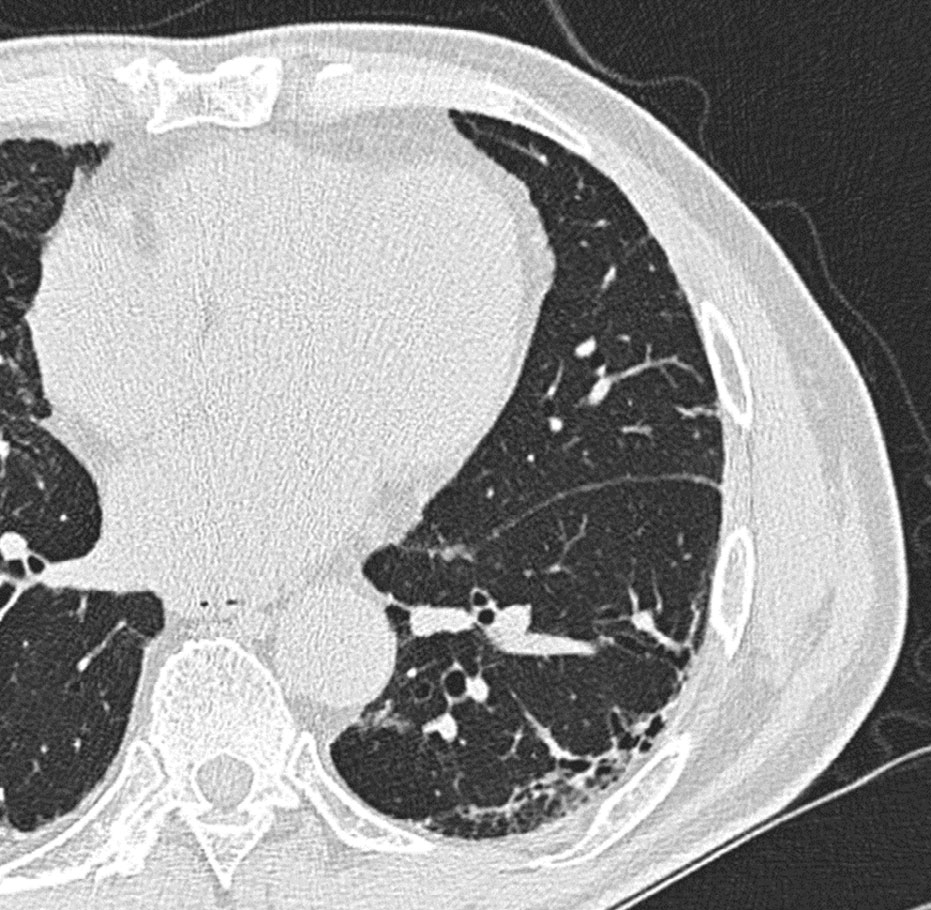
High‐resolution computed tomography image showing a typical example of pre‐existing interstitial lung disease (probable usual interstitial pneumonia pattern)

From 160 consecutive patients who were treated with ICIs plus chemotherapy, 28 patients who received radiotherapy in the chest, four patients who received sequential or concurrent chest radiotherapy, and three patients who previously received ICIs were excluded from the analysis. Therefore, the final analysis included 125 patients. The median duration of follow‐up was 13.1 months (range 0.4–27.7 months). The patient characteristics are provided in Table 1. The median age was 67 years (range, 25–84 years), 42 patients were female (33.6%), and 86 patients (68.8%) had a history of smoking. The histological types were adenocarcinoma in 92 patients (73.6%), squamous cell carcinoma in 17 patients (13.6%), and others in 16 patients (12.8%). Performance statuses of 0 or 1 were predominant. The proportions of patients with negative (<1%), low (1%–49%), and high PD‐L1 expression (≥50%) were approximately equal. Twenty patients (16.0%) had pre‐existing ILD on chest CT. A typical example of a pre‐existing ILD is shown in Figure 1. As for the CT criteria for usual interstitial pneumonia (UIP), nine patients (7.2%) were classified as having probable UIP, nine (7.2%) as indeterminate for UIP, and two (1.6%) as having alternative diagnosis. Concerning the treatment regimen, 60 patients (48.0%) received pembrolizumab with CBDCA plus PEM, five patients (4.0%) received pembrolizumab with CDDP plus PEM, two patients (1.6%) received pembrolizumab with CBDCA plus PTX, 21 patients (16.8%) received pembrolizumab with CBDCA plus nab‐PTX, 29 patients (23.2%) received atezolizumab with BEV plus CBDCA and PTX, and eight patients (6.4%) received atezolizumab with CBDCA plus nab‐PTX.

**TABLE 1:** Patient characteristics

Characteristics	*n* = 125 (%)
Age, years	
Median (range)	67 (25–84)
< 65	50 (40.0)
≥ 65	75 (60.0)
Sex	
Male	83 (66.4)
Female	42 (33.6)
Smoking status	
Current/former smoker	86 (68.8)
Never smoked	39 (31.2)
Performance status	
0	52 (41.6)
1	57 (45.6)
2–3	16 (12.8)
Clinical stage	
III	5 (4.0)
IV	85 (68.0)
Postoperative recurrence	35 (28.0)
Histology	
Adenocarcinoma	92 (73.6)
Squamous cell carcinoma	17 (13.6)
Others^a^	16 (12.8)
PD‐L1 status	
<1%	33 (26.4)
1–49%	42 (33.6)
≥50%	40 (32.0)
unknown	10 (8.0)
Pre‐existing ILD on chest CT	
Non‐ILD	105 (84.0)
Probable UIP	9 (7.2)
Indeterminate for UIP	9 (7.2)
Alternative diagnosis	2 (1.6)
Regimen	
CBDCA + PEM + pembrolizumab	60 (48.0)
CDDP + PEM + pembrolizumab	5 (4.0)
CBDCA + PTX + pembrolizumab	2 (1.6)
CBDCA + nab‐PTX + pembrolizumab	21 (16.8)
CBDCA + PTX + BEV + atezolizumab	29 (23.2)
CBDCA + nab‐PTX + atezolizumab	8 (6.4)

Abbreviations: BEV, bevacizumab; CBDCA, carboplatin; CT, computed tomography; ILD, interstitial lung disease; nab‐PTX, nanoparticle albumin‐bound paclitaxel; PD‐L1, programmed death‐ligand 1; PEM, pemetrexed; PTX, paclitaxel; UIP, usual interstitial pneumonia.

^a^ Eight patients had lung cancer not otherwise specified, three patients had large cell neuroendocrine carcinoma, three patients had sarcomatoid carcinoma, one patient had adenosquamous cell carcinoma, and one patient had adenoid cystic carcinoma.

### Incidence and predictive factors of pneumonitis

Seventeen patients (13.6%) experienced drug‐induced pneumonitis, including eight, four, and five cases of grade 1, grade 2, and grade 3 pneumonitis, respectively. In the analysis by pre‐existing ILD, seven of 105 patients (6.7%) with non‐ILD and 10 of 20 patients (50.0%) with pre‐existing ILD experienced pneumonitis (Table 2). Among them, pneumonitis developed in five of the nine patients (55.6%) with probable UIP and four of the nine patients (44.4%) were indeterminate for UIP.

**TABLE 2:** Incidence of drug‐induced pneumonitis

	All patients			Non‐ILD			Pre‐existing ILD		
	Total, n	All‐grade, n (%)	Grade ≥ 3, n (%)	Total, n	All‐grade, n (%)	Grade ≥ 3, n (%)	Total, n	All‐grade, n (%)	Grade ≥ 3, n (%)
	125	17 (13.6)	5 (4.0)	105	7 (6.7)	2 (1.9)	20	10 (50.0)	3 (15.0)
Pembrolizumab + platinum + PEM	65	12 (18.5)	5 (7.7)	57	5 (8.8)	2 (3.5)	8	7 (87.5)	3 (37.5)
Pembrolizumab + CBDCA + PTX/nab‐PTX	23	1 (4.4)	0	19	0	0	4	1 (25.0)	0
Atezolizumab + CBDCA + PTX + BEV	29	3 (10.3)	0	25	2 (8.0)	0	4	1 (25.0)	0
Atezolizumab + CBDCA + nab‐PTX	8	1 (12.5)	0	4	0	0	4	1 (25.0)	0

Abbreviations: BEV, bevacizumab; CBDCA, carboplatin; CDDP, cisplatin; ILD, interstitial lung disease; nab‐PTX, nanoparticle albumin‐bound paclitaxel; PEM, pemetrexed; PTX, paclitaxel.

The incidence rate of drug‐induced pneumonitis for each treatment regimen is presented in Table 2. In patients with pre‐existing ILD, pneumonitis developed in 87.5% of patients treated with pembrolizumab and platinum plus PEM, whereas the rate was 25.0% for other regimens. Grade ≥ 3 pneumonitis occurred only in pembrolizumab with platinum plus PEM (7.7%), two of 57 patients (3.5%) with non‐ILD and three of eight patients (37.5%) with pre‐existing ILD.

**FIGURE 2:**
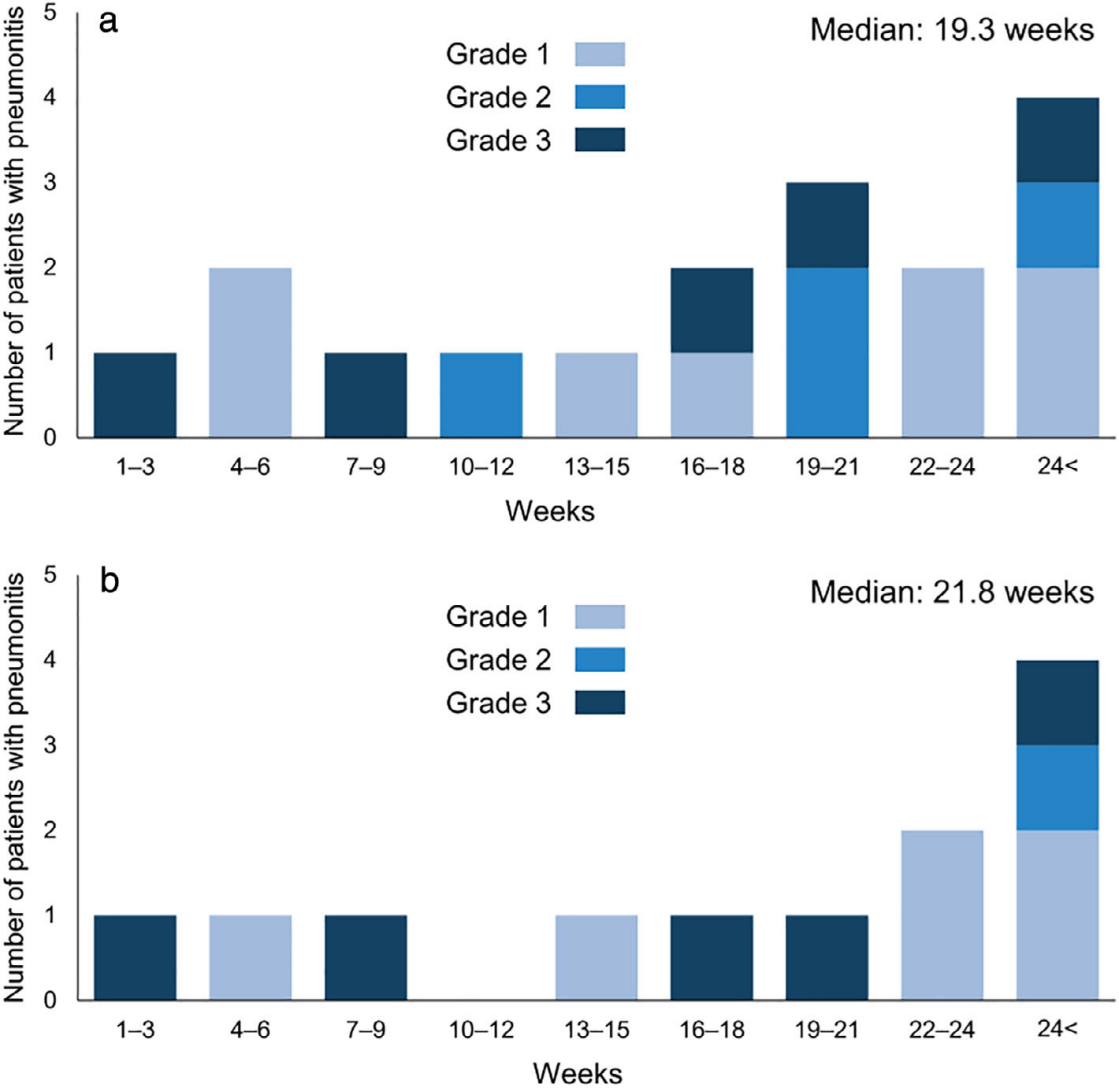
Common terminology criteria for adverse events (version 4.0) grade and time to onset of pneumonitis in all patients (a) and patients who received pembrolizumab and platinum plus pemetrexed (b)

The median onset time of pneumonitis was 19.3 weeks (range: 1.6–108.9 weeks). Of the 17 patients who experienced pneumonitis, four developed pneumonitis within 9 weeks from the start of ICI therapy plus chemotherapy, whereas four patients developed pneumonitis after ≥24 weeks (Figure 2a). In patients who received pembrolizumab and platinum plus PEM, the median onset time of pneumonitis was 21.8 weeks (range, 1.6–108.9 weeks) (Figure 2b). Of the 12 cases of pneumonitis, three developed within 9 weeks, and four developed after ≥24 weeks. Meanwhile, 7 of 12 patients developed pneumonitis after five or more doses of PEM.

In multivariate logistic regression analysis performed to analyze risk factors for pneumonitis, pre‐existing ILD (odds ratio = 19.07; 95% CI: 4.24–85.67, *p* = 0.0001) and PEM exposure (odds ratio = 5.67; 95% CI: 1.28–25.11, *p* = 0.022) were identified as risk factors (Table 3).

**TABLE 3:** Risk factors for pneumonitis after immune checkpoint inhibitors and chemotherapy by univariate and multivariate logistic regression analysis

		Univariate model			Multivariate model		
		OR	(95% CI)	*p*‐value	OR	(95% CI)	*p*‐value
Age, years	≥65 vs. <65	3.60	(0.98–13.24)	0.054	2.40	(0.56–10.27)	0.24
Sex	Female vs. male	0.38	(0.10–1.40)	0.15	0.69	(0.14–3.31)	0.64
Smoking status	Smoker vs. never smoked	2.33	(0.63–8.64)	0.205			
Performance status	≥2 vs. 0–1	1.57	(0.40–6.19)	0.52			
Histological subtype	Sq vs. non‐Sq	0.36	(0.044–2.90)	0.34			
Immune checkpoint inhibitors	Atezolizumab vs. pembrolizumab	0.70	(0.21–2.31)	0.56			
Chemotherapy	PEM vs. PTX/nab‐PTX	2.49	(0.82–7.55)	0.11	5.67	(1.28–25.11)	0.022
Bevacizumab	Yes vs. no	0.68	(0.18–2.54)	0.56			
Pre‐existing ILD	Yes vs. no	14.00	(4.37–44.86)	<0.0001	19.07	(4.24–85.67)	0.0001
PD‐L1 status	≥1% vs. <1%	2.06	(0.55–7.70)	0.28			
	≥50% vs. <50%	1.38	(0.48–3.95)	0.55			

Abbreviations: CI, confidence interval; ILD, interstitial lung disease; nab‐PTX, nanoparticle albumin‐bound paclitaxel; PEM, pemetrexed; PTX, paclitaxel; PD‐L1, programmed death‐ligand 1; OR, odds ratio; Sq, squamous cell carcinoma.

### Comparison of PFS and OS

**FIGURE 3:**
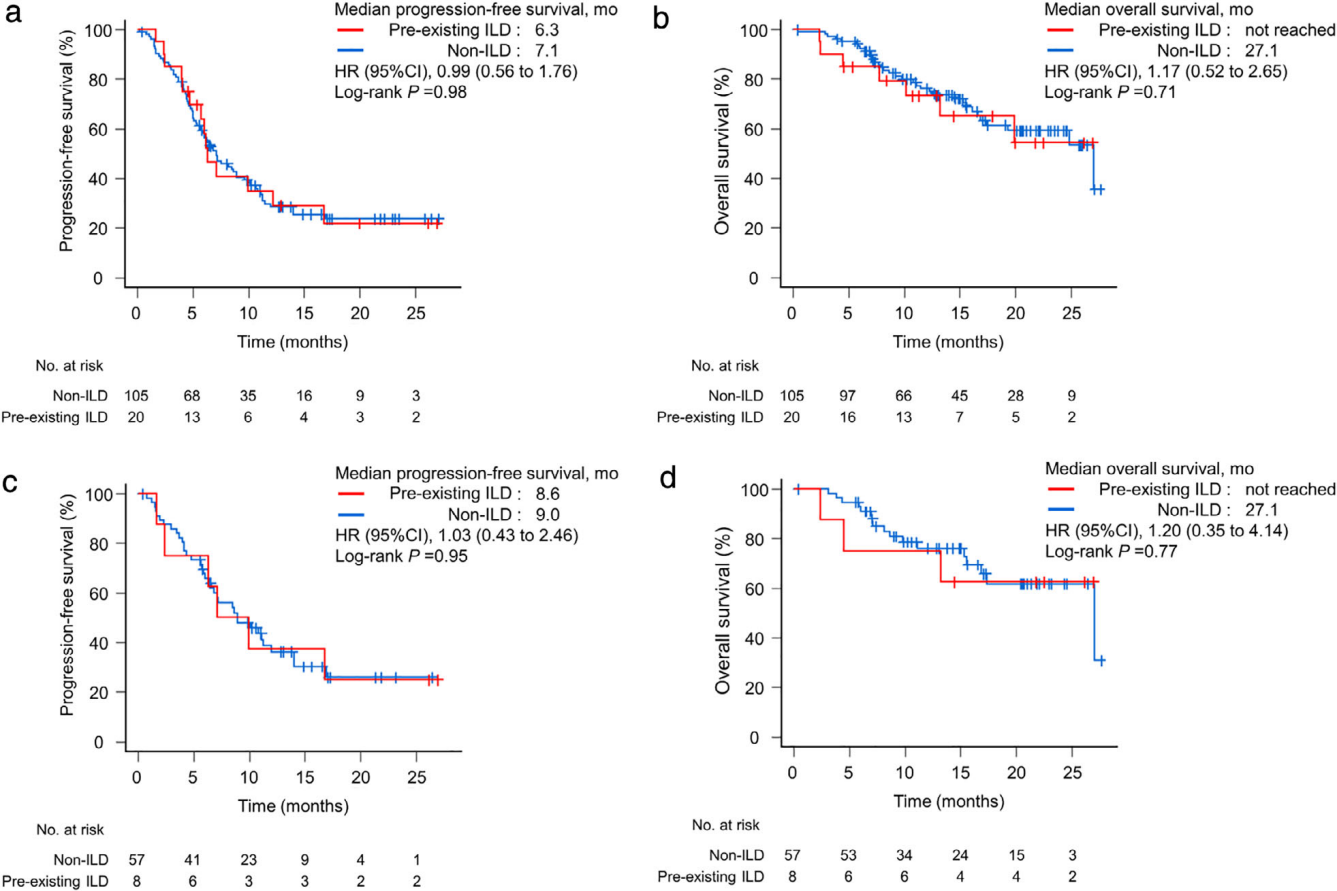
Kaplan–Meier survival curves of progression‐free survival and overall survival in all patients (a and b) and patients who received pembrolizumab and platinum plus pemetrexed (c and d)

In all patients, median PFS was 7.1 months (95% CI:  17.4–not reached), and median OS was 27.1 months (95% CI: 10.2–not reached). The median PFS and OS for each regimen are presented in Supplementary Figure S1 A and B. There were no significant differences in PFS and OS between the pre‐existing ILD and non‐ILD groups. Median PFS was 6.3 months in the pre‐existing ILD group and 7.1 months in the non‐ILD group (HR = 0.99; 95% CI: 0.56–1.76, *p* = 0.98, log‐rank, Figure 3a), and median OS was not reached in the pre‐existing ILD group and 27.1 months in the non‐ILD group (HR = 1.17; 95% CI: 0.52–2.65, *p* = 0.71, log‐rank, Figure 3b). In patients who received pembrolizumab and platinum plus PEM, the median PFS times in the pre‐existing‐ILD and non‐ILD groups were 8.6 and 9.0 months, respectively (HR = 1.03; 95% CI:  0.43–2.46, *p* = 0.95, log‐rank, Figure 3c), and the median OS times in these groups were not reached and 27.1 months, respectively (HR = 1.20; 95% CI: 0.35–4.14, *p* = 0.77, log‐rank, Figure 3d).

## DISCUSSION

This study revealed that pre‐existing ILD is a risk factor for pneumonitis induced by ICI therapy and chemotherapy in patients with NSCLC. In phase III clinical trials, the incidence of pneumonitis in patients with NSCLC treated with PD‐1 inhibitor monotherapy was reported to be approximately 3%–6%.^1^, ^2^, ^3^, ^4^, ^5^ On the contrary, according to reports based on real‐world data, the incidence rate of ICI‐induced pneumonitis tended to be high at approximately 10%–20%.^11^, ^12^, ^13^, ^14^ This increased rate of drug‐induced pneumonitis may be because the real‐world data included patients who did not meet the eligibility criteria of clinical trials, such as patients with pre‐existing ILD, radiation pneumonitis, poor performance status, and collagen disease.

Based on the results of several clinical trials, the combination of ICI therapy plus chemotherapy has become the standard of care for patients with NSCLC in the first‐line setting. However, the addition of ICIs to chemotherapy has directed attention toward various irAEs. In particular, ICI therapy plus chemotherapy is associated with a higher risk of pneumonitis than chemotherapy alone.^16^ In the present study, we retrospectively analyzed patients who received ICIs plus chemotherapy in clinical practice, and the incidence rate of pneumonitis was 13.6%. In cancer chemotherapy, pre‐existing ILD is generally considered a risk factor for drug‐induced pneumonitis. Previous studies reported a significant increase in the risk of drug‐induced pneumonitis associated with cytotoxic chemotherapy in patients with lung cancer and pre‐existing ILD, with incidence rates of approximately 10%–20%.^19^, ^20^, ^21^, ^22^, ^23^ Recently, multiple reports described the development of ICI‐induced pneumonitis and its risk factors.^12^, ^14^, ^15^ In our previous retrospective analysis of PD‐1 inhibitor monotherapy for NSCLC, pre‐existing ILD was identified as a risk factor for ICI‐induced pneumonitis, and the frequency of pneumonitis increased from 5.8% in the non‐ILD group to 35.1% in the pre‐existing ILD group.^12^ Of particular note, UIP may be the most notable risk factor, with 55.6% of patients with UIP experiencing pneumonitis in this study. This study did not include patients with UIP on chest CT; however, 6.6% of patients with non‐ILD and 50.0% of patients with pre‐existing ILD developed ICI/chemotherapy‐induced pneumonitis. It is considered that pre‐existing ILD is a risk factor for ICI/chemotherapy‐induced pneumonitis even when the UIP pattern is not included.

In multivariate analysis, PEM was also considered a risk factor for drug‐induced pneumonitis. Of the 65 patients who received pembrolizumab with platinum plus PEM, 12 (18.5%) developed pneumonitis. It is noteworthy that seven of eight patients (87.5%) with pre‐existing ILD who received pembrolizumab with platinum plus PEM experienced pneumonitis. PEM, similarly as other cytotoxic chemotherapeutic agents, can cause pneumonitis with a reported incidence of 1.6%–2.6% according to post‐marketing surveillance studies.^24^, ^25^ It has also been reported that pre‐existing ILD is a risk factor for the development of PEM‐induced pneumonitis.^25^ Although PEM or PTX/nab‐PTX was used in combination with ICIs, it is unclear whether the two drugs carry different risks of pneumonitis, especially among patients with pre‐existing ILD. Two phase II trials examined the efficacy and safety of CBDCA plus PTX or CBDCA plus nab‐PTX in patients with NSCLC and pre‐existing ILD, and the frequency of developing pneumonitis ranged from 4.3%–5.6%.^26^, ^27^ Conversely, no prospective trials have examined PEM in patients with pre‐existing ILD. Therefore, more safety data are available for PTX/nab‐PTX than for PEM regarding the risk of drug‐induced pneumonitis in patients with NSCLC and pre‐existing ILD. Increasing the number of PEM doses because of maintenance treatment may also have affected the onset of pneumonitis. PTX/nab‐PTX was administered for up to four cycles, whereas PEM treatment was continued until disease progression or toxicity. Many cytotoxic anticancer agents have been reported to promote pulmonary fibrosis with repeated administration, resulting in a gradual decrease in the diffusing capacity of the lungs and an increase in lung fibrosis findings on chest CT.^28^, ^29^ In the present study, seven of 12 patients who experienced pneumonitis after administration of pembrolizumab and platinum plus PEM received PEM more than five times before the onset of pneumonitis. Repeated administration of PEM may have promoted fibrosis of the lungs, creating an environment in which ICI‐induced pneumonitis is likely to develop.

The decision regarding ICI use in patients with NSCLC and pre‐existing ILD is an important concern. Pneumonitis is a serious irAE that can make it difficult to continue treatment, and in some cases, a long‐term continuous treatment effect is observed even after treatment discontinuation. In the present study, there were no significant differences in PFS and OS between the pre‐existing ILD and non‐ILD groups. Patients with pre‐existing ILD are at increased risk of drug‐induced pneumonitis, whereas ICI administration may provide a durable response. Although pembrolizumab with platinum plus PEM induced drug‐induced pneumonitis in 87.5% of patients with pre‐existing ILD, there were no significant differences in PFS and OS between patients with and without ILD. Conversely, steroid therapy is effective against ICI‐induced pneumonitis, providing high remission and low mortality rates.^30^, ^31^ Therefore, we consider that ICIs are contraindicated in patients with pre‐existing ILD, although high‐risk cases require constant attention regarding pneumonitis and detailed explanations to the patient before use.

The present study had several limitations. First, this was a retrospective study conducted in a single center, and the sample size was small. Second, the analysis population did not include patients with the UIP pattern; therefore, the incidence rate of pneumonitis in patients with pre‐existing UIP pattern ILD is unknown. Third, drug‐induced pneumonitis has been found to be common in Japanese patients, which may have influenced the results of this study.^20^, ^32^ Fourth, high‐resolution CT (HR‐CT) was not available for analysis because this type of imaging is not routinely performed at our institution despite the Official ATS/ERS/JRS/ALAT Clinical Practice Guideline recommending classifying image patterns on chest HR‐CT images.^17^ None of the study patients had a UIP pattern with honeycombing; however, it is important to note that HR‐CT data are needed to accurately classify the patterns of UIP on imaging and determine their incidence.

In conclusion, our study identified pre‐existing ILD on chest CT and PEM treatment as risk factors for drug‐induced pneumonitis in patients who received with ICIs plus chemotherapy. It should be noted that the administration of PEM plus ICIs to patients with pre‐existing ILD carries a high risk of pneumonitis. However, because the therapeutic effect of ICIs plus chemotherapy is not inferior even in patients with pre‐existing ILD, treatment should be selected in consideration of the risk of pneumonitis and the survival benefit.

## CONFLICT OF INTEREST

TY reported receiving personal fees from Ono Pharmaceutical, Chugai Pharmaceutical, Eli Lilly, Taiho Pharmaceutical, AstraZeneca, MSD, and Bristol‐Meyers Squibb outside the submitted work. JS reported receiving personal fees from AstraZeneca, Bristol‐Myers Squibb, Ono Pharmaceutical, Chugai Pharmaceutical, and MSD. YO reported receiving personal fees from AstraZeneca, Daiichi Sankyo, Ono Pharmaceutical, Chugai Pharmaceutical, Taiho Pharmaceutical, Eli Lilly, Bristol‐Myers Squibb, and Amgen outside the submitted work. YF reported receiving grant from Chugai Pharmaceutical, and personal fees from Astra Zeneca, Daiichi Sankyo, ONO Pharmaceutical, Otsuka Pharmaceutical, Novartis, and Yakult outside the submitted work. All other authors have no conflicts of interest.

## Supporting information


**Figure S1** Kaplan–Meier survival curves of progression‐free survival (A) and overall survival (B) for each regimen. PEM, pemetrexed; CBDCA, carboplatin; PTX, paclitaxel; nab‐PTX, nanoparticle albumin‐bound paclitaxel; BEV, bevacizumab.

## References

[1] Brahmer J , Reckamp KL , Baas P , et al. Nivolumab versus docetaxel in advanced squamous‐cell non‐small‐cell lung cancer. N Engl J Med. 2015;373(2):123–35. 26028407 10.1056/NEJMoa1504627 PMC4681400

[2] Borghaei H , Paz‐Ares L , Horn L , et al. Nivolumab versus docetaxel in advanced nonsquamous non‐small‐cell lung cancer. N Engl J Med. 2015;373(17):1627–39. 26412456 10.1056/NEJMoa1507643 PMC5705936

[3] Herbst RS , Baas P , Kim D‐W , et al. Pembrolizumab versus docetaxel for previously treated, PD‐L1‐positive, advanced non‐small‐cell lung cancer (KEYNOTE‐010): a randomised controlled trial. Lancet. 2016;387(10027):1540–50. 26712084 10.1016/S0140-6736(15)01281-7

[4] Reck M , Rodríguez‐Abreu D , Robinson AG , et al. Pembrolizumab versus chemotherapy for PD‐L1‐positive non‐small‐cell lung cancer. N Engl J Med. 2016;375(19):1823–33. 27718847 10.1056/NEJMoa1606774

[5] Rittmeyer A , Barlesi F , Waterkamp D , et al. Atezolizumab versus docetaxel in patients with previously treated non‐small‐cell lung cancer (OAK): a phase 3, open‐label, multicentre randomised controlled trial. Lancet. 2017;389(10066):255–65. 27979383 10.1016/S0140-6736(16)32517-X PMC6886121

[6] Langer CJ , Gadgeel SM , Borghaei H , et al. Carboplatin and pemetrexed with or without pembrolizumab for advanced, non‐squamous non‐small‐cell lung cancer: a randomised, phase 2 cohort of the open‐label KEYNOTE‐021 study. Lancet Oncol. 2016;17(11):1497–508. 27745820 10.1016/S1470-2045(16)30498-3 PMC6886237

[7] Gandhi L , Rodríguez‐Abreu D , Gadgeel S , et al. Pembrolizumab plus chemotherapy in metastatic non‐small‐cell lung cancer. N Engl J Med. 2018;378(22):2078–92. 29658856 10.1056/NEJMoa1801005

[8] Paz‐Ares L , Luft A , Vicente D , et al. Pembrolizumab plus chemotherapy for squamous non‐small‐cell lung cancer. N Engl J Med. 2018;379(21):2040–51. 30280635 10.1056/NEJMoa1810865

[9] Socinski MA , Jotte RM , Cappuzzo F , et al. Atezolizumab for first‐line treatment of metastatic nonsquamous NSCLC. N Engl J Med. 2018;378(24):2288–301. 29863955 10.1056/NEJMoa1716948

[10] West H , McCleod M , Hussein M , et al. Atezolizumab in combination with carboplatin plus nab‐paclitaxel chemotherapy compared with chemotherapy alone as first‐line treatment for metastatic non‐squamous non‐small‐cell lung cancer (IMpower130): a multicentre, randomised, open‐label, phase 3 trial. Lancet Oncol. 2019;20(7):924–37. 31122901 10.1016/S1470-2045(19)30167-6

[11] Suzuki Y , Karayama M , Uto T , et al. Assessment of immune‐related interstitial lung disease in patients with NSCLC treated with immune checkpoint inhibitors: a multicenter prospective study. J Thorac Oncol. 2020;15(8):1317–27. 32289515 10.1016/j.jtho.2020.04.002

[12] Yamaguchi T , Shimizu J , Hasegawa T , et al. Pre‐existing pulmonary fibrosis is a risk factor for anti‐PD‐1‐related pneumonitis in patients with non‐small cell lung cancer: a retrospective analysis. Lung Cancer. 2018;125:212–7. 30429022 10.1016/j.lungcan.2018.10.001

[13] Fujimoto D , Yoshioka H , Kataoka Y , et al. Efficacy and safety of nivolumab in previously treated patients with non‐small cell lung cancer: a multicenter retrospective cohort study. Lung Cancer. 2018;119:14–20. 29656747 10.1016/j.lungcan.2018.02.017

[14] Tamiya A , Tamiya M , Nakahama K , et al. Correlation of radiation pneumonitis history before Nivolumab with onset of interstitial lung disease and progression‐free survival of patients with pre‐treated advanced non‐small cell lung cancer. Anticancer Res. 2017;37(9):5199–205. 28870955 10.21873/anticanres.11943

[15] Suresh K , Voong KR , Shankar B , et al. Pneumonitis in non‐small cell lung cancer patients receiving immune checkpoint immunotherapy: incidence and risk factors. J Thorac Oncol. 2018;13(12):1930–9. 30267842 10.1016/j.jtho.2018.08.2035

[16] Chen X , Zhang Z , Hou X , et al. Immune‐related pneumonitis associated with immune checkpoint inhibitors in lung cancer: a network meta‐analysis. J Immunother Cancer. 2020;8(2):e001170. 32863271 10.1136/jitc-2020-001170 PMC7462235

[17] Raghu G , Remy‐Jardin M , Myers JL , et al. Diagnosis of idiopathic pulmonary fibrosis. An official ATS/ERS/JRS/ALAT clinical practice guideline. Am J Respir Crit Care Med. 2018;198(5):e44–68. 30168753 10.1164/rccm.201807-1255ST

[18] Common Terminology Criteria for Adverse Events (CTCAE), Version 5.0 Published: November 27, 2017. U.S. Department of Health and Human Services, National Institute of Health National Cancer Institute. [cited 2021 December 15]. Available from: https://ctep.cancer.gov/protocolDevelopment/electronic_applications/ctc.htm.

[19] Togashi Y , Masago K , Handa T , et al. Prognostic significance of preexisting interstitial lung disease in Japanese patients with small‐cell lung cancer. Clin Lung Cancer. 2012;13(4):304–11. 22169479 10.1016/j.cllc.2011.11.001

[20] Kudoh S , Kato H , Nishiwaki Y , et al. Interstitial lung disease in Japanese patients with lung cancer: a cohort and nested case‐control study. Am J Respir Crit Care Med. 2008;177(12):1348–57. 18337594 10.1164/rccm.200710-1501OC

[21] Usui K , Tanai C , Tanaka Y , Noda H , Ishihara T . The prevalence of pulmonary fibrosis combined with emphysema in patients with lung cancer. Respirology. 2011;16(2):326–31. 21114711 10.1111/j.1440-1843.2010.01907.x

[22] Tomassetti S , Gurioli C , Ryu JH , et al. The impact of lung cancer on survival of idiopathic pulmonary fibrosis. Chest. 2015;147(1):157–64. 25166895 10.1378/chest.14-0359

[23] Fujimoto D , Kato R , Morimoto T , et al. Characteristics and prognostic impact of pneumonitis during systemic anti‐cancer therapy in patients with advanced non‐small‐cell lung cancer. PLoS One. 2016;11(12):e0168465. 28006019 10.1371/journal.pone.0168465 PMC5179067

[24] Kuribayashi K , Voss S , Nishiuma S , et al. Safety and effectiveness of pemetrexed in patients with malignant pleural mesothelioma based on all‐case drug‐registry study. Lung Cancer. 2012;75(3):353–9. 21890228 10.1016/j.lungcan.2011.08.002

[25] Tomii K , Kato T , Takahashi M , et al. Pemetrexed‐related interstitial lung disease reported from post marketing surveillance (malignant pleural mesothelioma/non‐small cell lung cancer). Jpn J Clin Oncol. 2017;47(4):350–6. 28158568 10.1093/jjco/hyx010

[26] Minegishi Y , Sudoh J , Kuribayasi H , et al. The safety and efficacy of weekly paclitaxel in combination with carboplatin for advanced non‐small cell lung cancer with idiopathic interstitial pneumonias. Lung Cancer. 2011;71(1):70–4. 20493578 10.1016/j.lungcan.2010.04.014

[27] Kenmotsu H , Yoh K , Mori K , et al. Phase II study of nab‐paclitaxel + carboplatin for patients with non‐small‐cell lung cancer and interstitial lung disease. Cancer Sci. 2019;110(12):3738–45. 31608537 10.1111/cas.14217 PMC6890441

[28] Dimopoulou I , Galani H , Dafni U , Samakovii A , Roussos C , Dimopoulos MA . A prospective study of pulmonary function in patients treated with paclitaxel and carboplatin. Cancer. 2002;94(2):452–8. 11900231 10.1002/cncr.10182

[29] Yumuk PF , Kefeli U , Ceyhan B , et al. Pulmonary toxicity in patients receiving docetaxel chemotherapy. Med Oncol. 2010;27(4):1381–8. 20035385 10.1007/s12032-009-9391-9

[30] Kato T , Masuda N , Nakanishi Y , et al. Nivolumab‐induced interstitial lung disease analysis of two phase II studies patients with recurrent or advanced non‐small‐cell lung cancer. Lung Cancer. 2017;104:111–8. 28212992 10.1016/j.lungcan.2016.12.016

[31] Yamaguchi O , Kaira K , Shinomiya S , et al. Pre‐existing interstitial lung disease does not affect prognosis in non‐small cell lung cancer patients with PD‐L1 expression ≥50% on first‐line pembrolizumab. Thorac Cancer. 2021;12(3):304–13. 33185333 10.1111/1759-7714.13725 PMC7862785

[32] Hida T , Kaji R , Satouchi M , et al. Atezolizumab in Japanese patients with previously treated advanced non‐small‐cell lung cancer: a subgroup analysis of the phase 3 OAK study. Clin Lung Cancer. 2018;19(4):e405–e15. 29525239 10.1016/j.cllc.2018.01.004

