# Supplementary material for: Risk factors for pneumonitis in patients with non‐small cell lung cancer treated with immune checkpoint inhibitors plus chemotherapy: A retrospective analysis
Source: Thorac Cancer. 2022 Jan 19;13(5):724–31. doi: 10.1111/1759-7714.14308 (PMC8888158; doi:10.1111/1759-7714.14308)
Supplement: Supplementary file 1 — Figure S1 Kaplan–Meier survival curves of progression‐free survival (A) and overall survival (B) for each regimen. PEM, pemetrexed; CBDCA, carboplatin; PTX, paclitaxel; nab‐PTX, nanoparticle albumin‐bound paclitaxel; BEV, bevacizumab. [file TCA-13-724-s001.pdf]

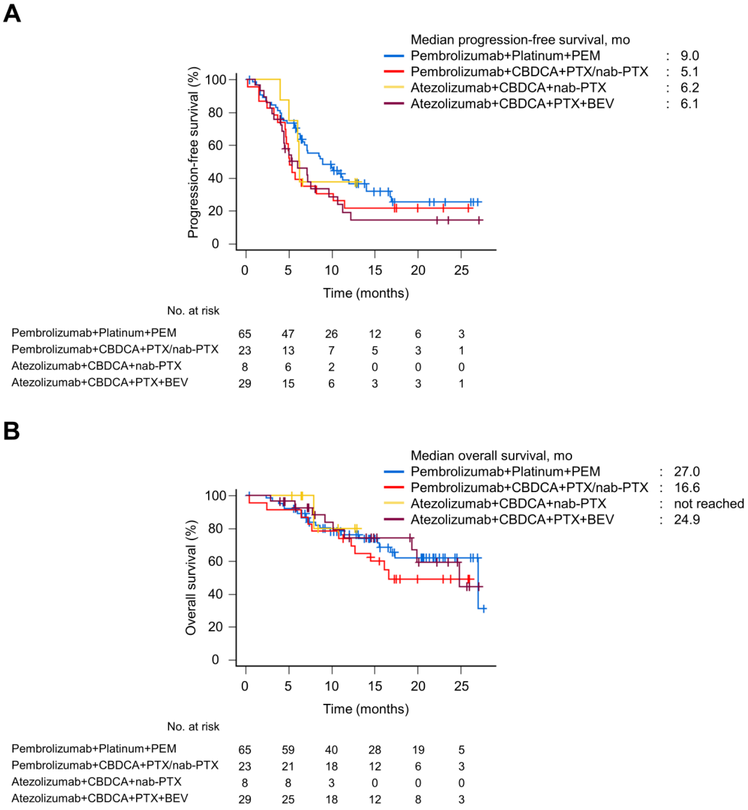
A
Median progression-free survival, mo
Pembrolizumab+Platinum+PEM : 9.0
Pembrolizumab+CBDCA+PTX/nab-PTX : 5.1
Atezolizumab+CBDCA+nab-PTX : 6.2
Atezolizumab+CBDCA+PTX+BEV : 6.1
Progression-free survival (%)
100
80
60
40
20
0
0
5
10
15
20
25
Time (months)
No. at risk
Pembrolizumab+Platinum+PEM 65 47 26 12 6 3
Pembrolizumab+CBDCA+PTX/nab-PTX 23 13 7 5 3 1
Atezolizumab+CBDCA+nab-PTX 8 6 2 0 0 0
Atezolizumab+CBDCA+PTX+BEV 29 15 6 3 3 1
B
Median overall survival, mo
Pembrolizumab+Platinum+PEM : 27.0
Pembrolizumab+CBDCA+PTX/nab-PTX : 16.6
Atezolizumab+CBDCA+nab-PTX : not reached
Atezolizumab+CBDCA+PTX+BEV : 24.9
Overall survival (%)
100
80
60
40
20
0
0
5
10
15
20
25
Time (months)
No. at risk
Pembrolizumab+Platinum+PEM 65 59 40 28 19 5
Pembrolizumab+CBDCA+PTX/nab-PTX 23 21 18 12 6 3
Atezolizumab+CBDCA+nab-PTX 8 8 3 0 0 0
Atezolizumab+CBDCA+PTX+BEV 29 25 18 12 8 3
